# Accurate classification of carotid endarterectomy indication using physician claims and hospital discharge data

**DOI:** 10.1186/s12913-022-07614-1

**Published:** 2022-03-22

**Authors:** Stephen van Gaal, Arshia Alimohammadi, Amy Y. X. Yu, Mohammad Ehsanul Karim, Wei Zhang, Jason M. Sutherland

**Affiliations:** 1 Faculty of Medicine, University of British Columbia, 8161-2775 Laurel Street, Vancouver, BC V5Z1M9 Canada; 2 Department of Medicine (Neurology), University of Toronto, Toronto, Canada; 3 Hurvitz Brain Sciences Program, Sunnybrook Health Sciences Centre, Toronto, Ontario Canada; 4 School of Population and Public Health, University of British Columbia, Centre for Health Evaluation and Outcome Sciences, Vancouver, Canada; 5 Centre for Health Services and Policy Research, University of British Columbia, Vancouver, Canada

**Keywords:** Machine learning, Administrative data, Carotid endarterectomy, Statistical model

## Abstract

**Background and purpose:**

Studies of carotid endarterectomy (CEA) require stratification by symptomatic vs asymptomatic status because of marked differences in benefits and harms. In administrative datasets, this classification has been done using hospital discharge diagnosis codes of uncertain accuracy. This study aims to develop and evaluate algorithms for classifying symptomatic status using hospital discharge and physician claims data.

**Methods:**

A single center’s administrative database was used to assemble a retrospective cohort of participants with CEA. Symptomatic status was ascertained by chart review prior to linkage with physician claims and hospital discharge data. Accuracy of rule-based classification by discharge diagnosis codes was measured by sensitivity and specificity. Elastic net logistic regression and random forest models combining physician claims and discharge data were generated from the training set and assessed in a test set of final year participants. Models were compared to rule-based classification using sensitivity at fixed specificity.

**Results:**

We identified 971 participants undergoing CEA at the Vancouver General Hospital (Vancouver, Canada) between January 1, 2008 and December 31, 2016. Of these, 729 met inclusion/exclusion criteria (*n* = 615 training, *n* = 114 test). Classification of symptomatic status using hospital discharge diagnosis codes was 32.8% (95% CI 29–37%) sensitive and 98.6% specific (96–100%). At matched 98.6% specificity, models that incorporated physician claims data were significantly more sensitive: elastic net 69.4% (59–82%) and random forest 78.8% (69–88%).

**Conclusion:**

Discharge diagnoses were specific but insensitive for the classification of CEA symptomatic status. Elastic net and random forest machine learning algorithms that included physician claims data were sensitive and specific, and are likely an improvement over current state of classification by discharge diagnosis alone.

**Supplementary Information:**

The online version contains supplementary material available at 10.1186/s12913-022-07614-1.

## Introduction

Historical evidence supports asymptomatic [1] (primary prevention) and symptomatic [2] (secondary prevention) indications for carotid endarterectomy (CEA), but there is equipoise for the benefit of asymptomatic intervention in the context of contemporary medical therapy [3]. If the ongoing CREST-2 study [4] fails to demonstrate the benefit of primary prevention CEA beyond medical therapy, then studies of the appropriate use of CEA by symptomatic status are expected to follow. Administrative data is likely to be used for these studies, given its low cost and population-level scope. Whether these studies will be valid will entirely depend on the accuracy of administrative data for classifying CEA symptomatic status. Classification of symptomatic status is also critical for the construction of administrative data indicators for CEA quality, including: (1) timeliness: a widely-accepted two-week time target is applicable only to secondary prevention [2] and (2) safety: acceptable rates of peri-operative morbidity and mortality for primary prevention are lower than rates acceptable for secondary prevention [5].

Using a systematic search strategy, we identified studies that used administrative data to classify the symptomatic status of patients who had undergone CEA (Supplementary Table 1). All studies identified by our search classified patients by rule using hospital discharge diagnosis. Only one group studied the performance of this classification relative to a gold standard chart review population, determining sensitivity 36.6% and specificity 93.1% [6]. Consistent with an insensitive measure, the rates for symptomatic intervention were generally much lower in administrative-data based cohorts (2.7–30.1%) [6–28] than in comparable registry cohorts relying on manual classification (CEA 30.7–43.5%) [28–32]. Rule-based classification by discharge diagnosis will likely yield ‘asymptomatic’ cohorts with many symptomatic patients, threatening the validity of studies that use this technique.

In this study, we aimed to improve upon the current rule-based method for classifying CEA symptomatic status by: 1) augmenting discharge data with physician claims data, and 2) using elastic net logistic regression and random forest machine learning (ML) models. We developed our models using a cohort of patients undergoing CEA at a major tertiary center in Vancouver, Canada between 2008 and 2015, and validated them in a cohort undergoing CEA at the same institution in 2016.

## Method

### Study setting and cohort

The setting of the study was the Vancouver General Hospital, a quaternary centre with a mixed catchment of local urban and rural/remote referral populations. We defined our study cohort by querying our institution’s hospital discharge database for carotid revascularization codes (Canadian Classification of Interventions 1.JE.57, 1.JE.50, 1.JE.87) [23] between January 1, 2008 and December 31, 2016. For participants with multiple procedures during this time, only the earliest was included. We identified very few instances of carotid stenting, so these patients were excluded from our cohort. Other exclusion criteria included: indication unrelated to atherosclerotic secondary prevention, symptomatic status not documented, symptom onset outside of province, symptom onset in hospital, stroke as complication of procedure, or initial treatment with medical therapy (Supplementary Fig. 1).

### Data sources

Participants were linked with population-level datasets for physician claims (Medical Services Plan Payment Information File [33] and Consolidation File [34]), hospital discharges (Discharge Abstract Database [35]) and demographics. These are available as by-products of the setting’s single-payer healthcare system, with near-universal population coverage. An anonymized dataset was created for analyses. Raw data were accessible to the investigators. The data that support the findings of this study are available from Population Data BC, but restrictions apply to the availability of these data, which were used under license for the current study, and so are not publicly available. Data are however available from the authors upon reasonable request and with permission of Population Data BC.

### Data preparation

Claims and hospital discharges were trimmed to a window of 6 months pre-surgery (by definition, symptomatic status refers to events that happened within 6 months of surgery [2], and we did not wish to add noise from possible earlier events) and 7 days post-procedure (to reduce risk of excluding relevant physician claims based on claim date error). Duplicate physician claims, generated through the billing adjudication process, were removed. Physician claims diagnoses (ICD-9) were truncated at three characters, as per previously published methods [36]. All test set participants were allocated from the final year of data to maximize similarity to future data [37].

### Discharge variables

All ICD-9-CM diagnostic codes used in previous studies [6–28] were mapped to their ICD-10-CA equivalents, which was the discharge coding standard used throughout our study period (Table 1). We additionally examined all I6X diagnostic codes present in our dataset to ensure that no relevant diagnoses were missed. Other discharge variables included age, sex, in-metro residence, and admission type (emergent, urgent, or elective).

**Table 1:** Diagnosis cluster definitions for ICD-9 and ICD-10-CA

Diagnosis cluster	ICD-10-CA (5-character code)	ICD-9 (3-digit code)
Ischemic stroke	I63.X (except I63.6, venous thrombosis), I64 (stroke not specified as hemorrhage or infarction), I66.0–2,4,8–9 (cerebral arterial stenosis or occlusion without infarction; included even though these codes specifically exclude infarction)	434 (some codes exclude infarction, but these are rarely used in BC), 436 (acute cerebrovascular disease), 438 (late effects of cerebrovascular disease)
TIA	G45.X (except G45.3, amaurosis fugax)	435
Retinal	G45.3, H34.X (retinal vascular occlusion), H35.82 (retinal ischemia), H53.1 (transient visual loss)	362 (retinal disorders), 368 (visual disturbances)
Cerebral	G81.0 & G81.9 (hemiparesis), H53.4 & H53.9 (visual non-retinal), R29.5 (transient paralysis of limb), R29.8 (other neurological symptoms), R47.0, R47.1 & R7.8 (aphasia & dysarthria)	781 (neurological symptoms), 341 (demyelinating symptom, but frequently used in our sample). Omit 784 (embeds speech in context of ‘head and neck’)
Stenosis (asymptomatic)	I65.2, I65.3, I65.9 (stenosis of carotid or multiple arteries)	433 (some codes include infarction, but these are rarely used in BC)

### Physician claims variables

Claims data were used to create diagnosis and service variables. Diagnoses were defined by 3-character ICD-9 codes, which was the coding standard for physician claims used throughout the study period (in contrast to discharge ICD-10 coding). Reasoning that the predictive value of a diagnosis might vary depending on the person making it, we pre-specified select diagnosis by specialty combinations (e.g. TIA by neurologist vs. non-neurologist (Supplementary Table 2). Based on clinical experience, we expected that symptomatic patients would undergo symptom-specific evaluation, such as a CT head for a new central nervous system symptom, and that this would be reflected in claimed services. We believed that the occurrence of these services would likely prove to be reliable signals for symptomatic events. Services were defined by 5-character fee item codes, which were clustered into categories based on descriptions in the physician payment schedule (Supplementary Table 3).

We used a 4-step workflow to transform the many-to-one raw data into a one-to-one analytical dataset: (1) clustering – grouping similar service or diagnosis items together, such as ICD-9362 retinal disorders and 368 visual disturbance, (2) reduction – removing conceptual duplicates, such as the same diagnosis made by the same provider on different days, (3) weighting – assigning a value based on time from CEA, and (4) summarizing – collapsing data into a single row as either the sum or maximum value (Supplementary Fig. 2 presents a step-by-step illustration of these terms in the context of our data).

### Missing values

Given the original dataset, missing values in predictor data are not possible (conditions are either observed or not observed). Median imputation was used for participants missing symptom type (*N* = 6), which was used only to describe the study population.

### Outcome definitions

The gold standard for this study was symptomatic status as assessed by chart review (SVG, AA). Consultations, operative notes, and discharge summaries within 6 months of the intervention were reviewed. Interrater agreement for symptomatic status was measured using the Kappa statistic for two raters. Interrater conflicts were subsequently resolved by consensus.

### Comparison of model discrimination

The ML models used in this analysis yield continuous probability values, which can be easily used in receiver-operating characteristic curve (ROC) analysis for the measurement of discrimination. However, the rule-based diagnosis method yields a single binary class, which cannot be used in ROC analysis. As an alternative, for each ML model we calculated confidence intervals for sensitivity at a fixed specificity matching the specificity of the rule-based method. Specifically, if the ML model’s 95% CI for sensitivity excluded the measured sensitivity of the rule-based method, at the same margin of specificity, we took this as evidence for superior discrimination of the ML model. We believe that this sets up a reasonable like-to-like comparison between binary and continuous classifier types. In contrast, because all ML models generate continuous output, ML models are compared to each other using the area under the ROC curve (AUC).

### Sample size

Sensitivity is a binomial proportion, so the necessary sample size can be estimated by calculating the confidence interval of a comparison value [38]. As long as the true sensitivity of the comparison test is 0.6 or greater, a test set of *n* = 75 participants (25 cases / 50 controls) will have more than 95% power to exclude the previously reported sensitivity of 36% [6]. Assuming a test set of no larger than 25% of the total, a minimum of *n* = 300 participants is required.

### Models

We specified a set of five models of increasing complexity: (1) Rule_DX_: rule-based classification by hospital discharge diagnosis alone (current state method), (2) Logistic_HOSP_: elastic net logistic using hospital discharge variables, (3) Logistic_DX_: elastic net logistic adding physician claims diagnoses to hospital discharge variables, (4) Logistic_ALL_ elastic net logistic using all variables, and (5) Forest_ALL_: random forest using all variables. Elastic net and random forest models were selected because they incorporate automated variable selection, require a modest amount of data for training (e.g. in contrast to neural network) and include variable importance metrics for model interpretation. Random forest models are additionally advantaged by the automatic incorporation of interactions, whereas elastic net models are advantaged by linear relationships (random forest are binary).

Models were built and analyzed using a 4-step workflow: (1) model specification tuning using training set 10-fold 3-repeat cross-validation, (2) model performance assessment using training set 10-fold 10-repeat cross-validation, (3) final model fitting using the complete training set, and (4) final model performance measurement using 2000 bootstrapped test set samples. Model specifications were tuned using the tidymodels implementation of Bayesian grid search [39], using default values except where noted. Elastic net logistic regression models were built using glmnet [40], with tuning of mixture and penalty (controlling variable selection and overfitting control). Random forest models were built using ranger [41], with tuning of the number of variables randomly sampled as candidates at each split (specified range 2–20), minimum node size, and tree number (all controlling the complexity of the resulting algorithm).

### Sensitivity and AUC

Exact binomial confidence intervals for the sensitivity and specificity of Rule_DX_ were calculated using the combined test and training sets. To permit like-to-like comparison between the diagnosis only and ML methods, the sensitivity of ML models was assessed at the specificity observed for Rule_DX_. Training set sensitivity and AUC were calculated using cross-fold hold-outs, yielding mean and standard error. Test set sensitivity and AUC were assessed via bootstrapped empirical 95% confidence intervals.

### Calibration

Calibration was assessed was assessed by plot within the test set using LOESS-smoothed curves [42]. These curves were used to calculate the calibration intercept a (assessing whether the number of predicted events matches the number of observed events) and slope b_L_ (assessing the tendency for extreme or uninformative predictions) [43]. The unreliability index was then calculated, which evaluates H_0_: a = 0 and b_L_ = 1 using a single 2-df chi square test.

### Variable importance

Variable importance was assessed by standardized logistic regression coefficients and ranger’s random forest permutation importance.

### Statistical software and reproducibility

R version 3.6.1 was used for all analyses. A comprehensive list of packages is provided in the Data Supplement. Annotated statistical code has been included in the Data Supplement and is available in native format on request.

This study was approved by the University of British Columbia Clinical Research Ethics Board, with waiver for informed consent. All methods were performed in concordance with institutional ethics guidelines and regulations.

## Results

### Cohort and chart review

Of 971 unique participants identified by procedure code, 729 were included in the analytic data set. This dataset was split by year into train (year 2008–2015, *n* = 614) and test sets (year 2016, *n* = 114). Participant characteristics are summarized in Table 2. Between-rater agreement was very strong for symptomatic status (Kappa = 0.846, *n* = 21).

**Table 2:** Characteristics of included participants by train and test population, Vancouver, Canada, 2008–2016. *P*-values reported for t-test (age) and Fisher exact test (all other variables)

	Train	Test	*P*-value
N	615	114	
Data years	2008–2015	2016	
Age – mean (SD)	72.1 (9.1)	71.3 (9.7)	0.4
Female – N (%)	207 (33.7%)	33 (28.9%)	0.4
In metro – N (%)	482 (78.4%)	89 (78.1%)	1
Symptom type – N (%)			0.02
Asymptomatic	189 (30.7%)	29 (25.4%)	
Retinal	111 (18.0%)	22 (19.3%)	
TIA	198 (32.2%)	27 (23.7%)	
Stroke	117 (19.0%)	36 (31.6%)	

### Sensitivity

**Fig. 1:**
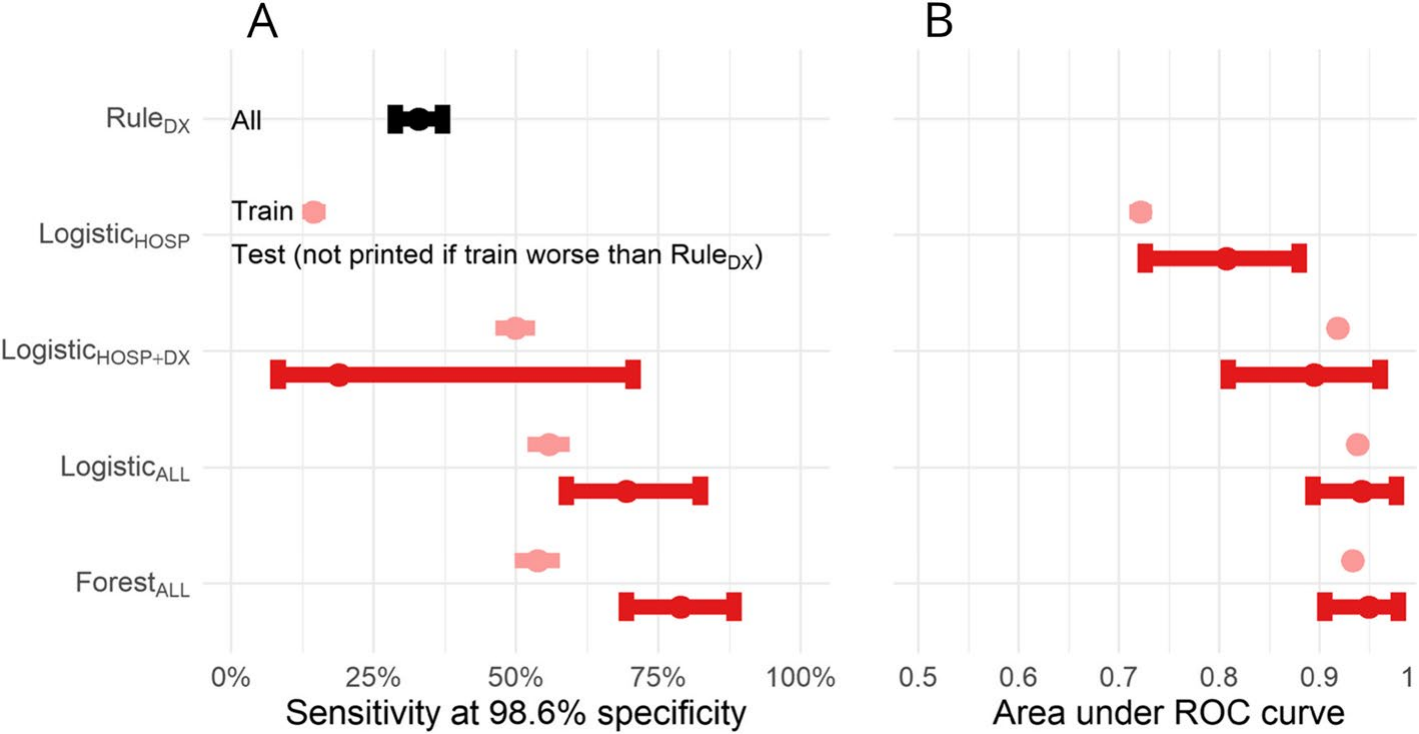
Model performance assessed by sensitivity at 98.6% specificity (panel **A**) and area under the receiver operating characteristic curve (panel **B**). The diagnosis-only rule-based method involves no parameter tuning, so it is reported for all participants. All other models are analysed by train and test population. Training results are calculated using cross-fold hold-outs and are plotted with mean and 95% confidence interval for standard error. Testing results are calculated using *n* = 2000 bootstrapped samples and are plotted by empiric 95% confidence interval. Area under the curve is not calculable for the rule-based diagnosis-only method

Model sensitivity is summarized in Fig. 1A. Within the combined train and test sets, Rule_DX_ was 32.8% (95% CI 29–37%) sensitive and 98.6% specific (96–100%). Within the train set, Logistic_DX_, Logistic_All_ and Forest_ALL_ were more sensitive than Rule_DX_ at the 98.6% specificity margin. Logistic_HOSP_ was less sensitive in the train set, so performance in the test set was not assessed. Test set confidence intervals for Logistic_All_ and Forest_All_ did not overlap with Rule_DX_, confirming the higher sensitivity of these models (95% CI 59–82% and 69–88%, respectively).

### Roc

**Fig. 2:**
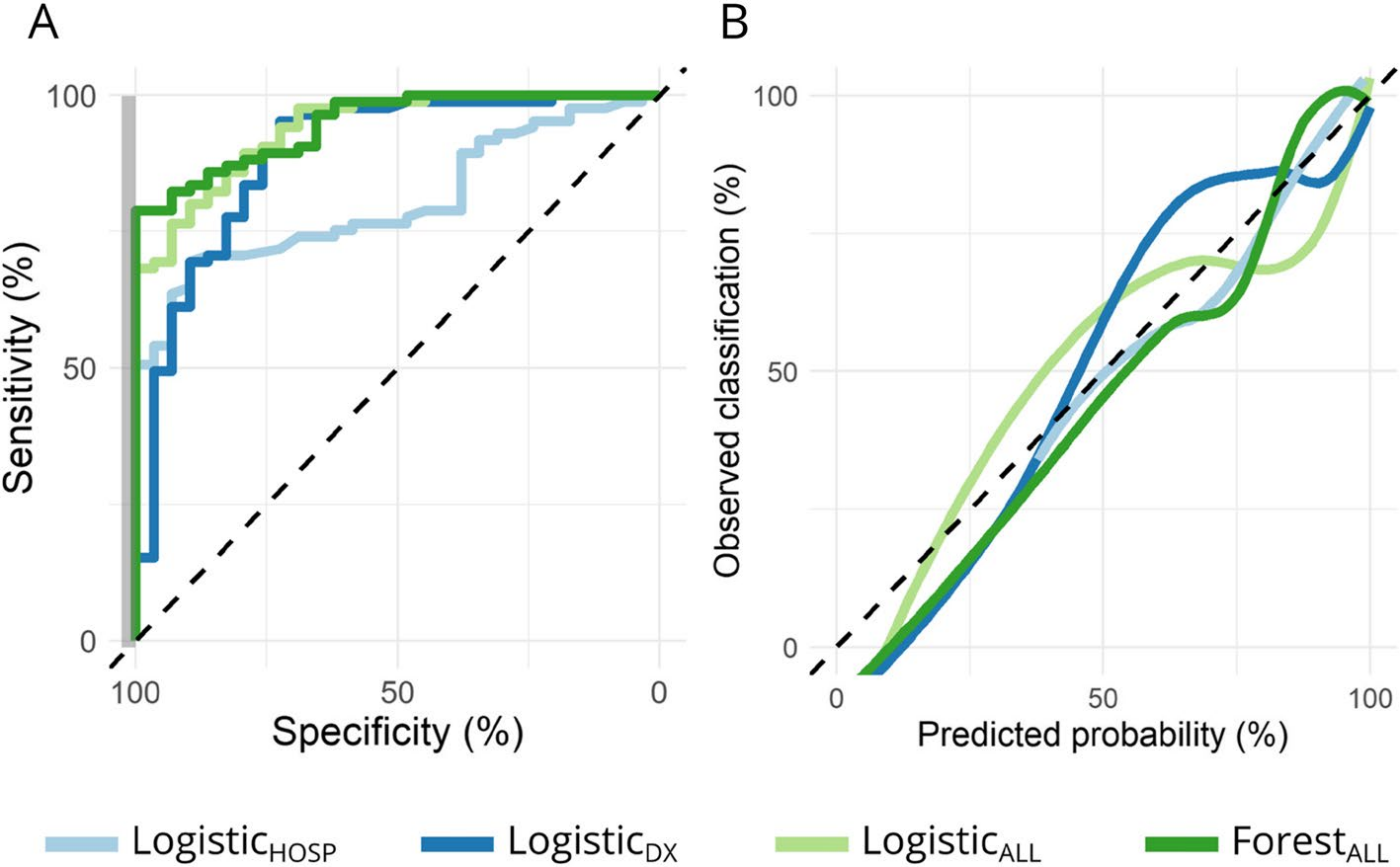
Model performance in the test set by receiver operator characteristic curve (panel **A**), observed vs. predicted probability calibration curve (panel **B**). In panel **A**, a grey bar is positioned at specificity 98–100%, highlighting the limited sensitivity of Logistic_DX_ at this margin

Model ROC AUC is summarized in Fig. 1B. Within the train set, mean AUC was highest for Logistic_ALL_ and Forest_ALL_ (0.938 ± 0.003 and 0.933 ± 0.003), intermediate for LogisticDX (0.918 ± 0.004) and lowest for Logistic_HOSP_ (0.722 ± 0.006). Within the test set, AUC 95% CI for Logistic_All_ and Forest_All_ (0.893–0.977 and 0.906–0.979) excluded Logistic_HOSP_ (0.726–0.879) and overlapped with Logistic_Dx_ (0.809–0.960). Test set ROC curves (Fig. 2A) highlight the discordance in AUC and sensitivity at the high specificity margin for Logistic_HOSP_ and Logistic_DX_.

### Calibration

LOESS-smoothed calibration curves are provided in Fig. 2B and summary statistics for calibration are provided in Supplementary Table 4. All logistic regression models approximated the identity line, consistent with good calibration-in-the-large (intercept) and weak calibration (slope). The unreliability index for the Forest_ALL_ calibration curve was marginally significant (*p*-value 0.053), indicating probable deviation from the identity line.

### Variable importance

**Fig. 3:**
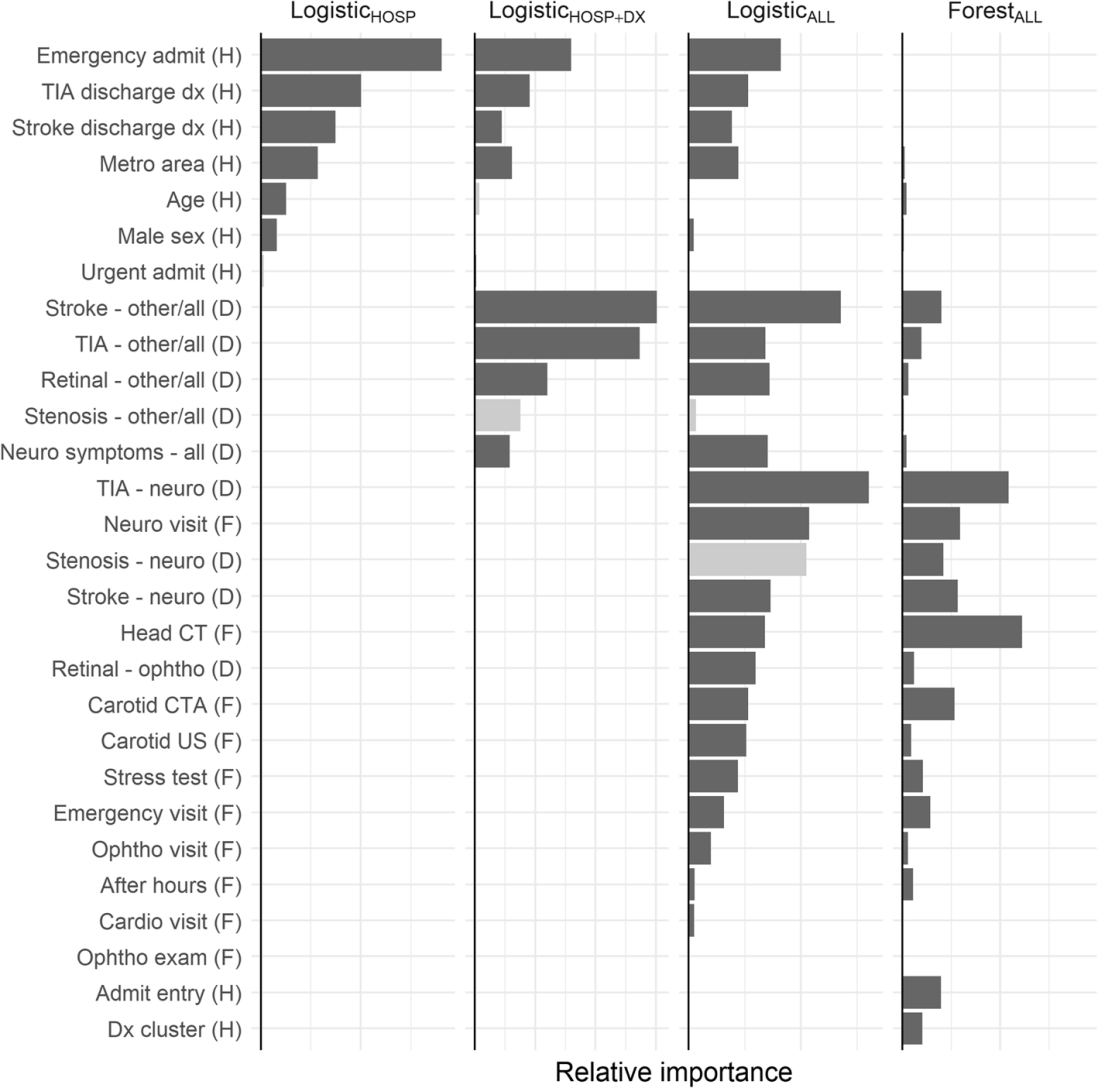
Standardized logistic regression model coefficients and random forest permutation importance for each model. Negative coefficients are shaded in light grey. Variables are sorted by importance in the first model in which they appear

Normalized logistic regression coefficients and random forest permutation importance metrics are presented in Fig. 3. Emergent hospital admission was weighted more heavily than discharge diagnoses. Physician diagnoses for stroke / TIA were heavily weighted, particularly those made by a neurologist. Fee items relating to the evaluation of probable ischemic symptoms, such as CT head, were moderately weighted.

## Discussion

In a cohort of participants with known CEA, rule-based classification by hospital discharge diagnosis was highly specific but insensitive for symptomatic status. The results of our study are concordant with a previous chart review [6] and likely explain implausibly low rates of symptomatic intervention observed in some administrative data studies [13, 18]. Given the available evidence, it is unlikely that this current state method will yield valid cohorts for population-level study of asymptomatic intervention.

We believe that the low sensitivity of diagnostic codes for symptomatic status is an expected side effect of Canadian discharge coding regulation. If a patient is initially admitted for a symptomatic event, such as an ischemic stroke, and undergoes unscheduled surgery on the same admission, the symptomatic event is correctly coded as either a most responsible diagnosis or preadmit comorbidity diagnosis type. These patients are likely to be correctly classified by a diagnosis-code only system. However, if the patient is admitted for scheduled surgery after their initial symptomatic event, then neither of these types can be used. It would be possible to code the ischemic event as a secondary diagnosis type, but this would also include ischemic events not relevant to the procedure. We do not see an obvious way to remedy this.

As an alternative to rule-based classification by discharge diagnosis, we explored the impact of increasingly complex ML models. To allow a direct comparison against rule-based classification, we evaluated all models by sensitivity at fixed high specificity. Our simplest model, elastic net with hospital discharge variables, performed no better by this metric in the training set and was not evaluated further. Adding physician claims diagnoses improved sensitivity in the training set but was inconsistently sensitive in the test set.

We hypothesized that two novel variable types might improve upon this result. First, we included a limited set of specialist-specific diagnoses, such as TIA diagnosed by neurologist, reasoning that these would be more predictive of true symptomatic status a diagnosis of TIA by a non-neurologist. Second, we identified a set of services, such as head CT, that might signal the investigation of a symptomatic event. Variables from both of these types were heavily weighted in both the logistic regression and random forest models, suggesting they are discriminating for symptomatic status. Performance of a head CT was especially important to the random forest model, reflecting its status as an essential investigation in the investigation of any central nervous system symptom. Incorporating these variables substantially improved model performance, in particular sensitivity at the high specificity margin. We believe that the gains in model performance justify the modest increase in data processing and model complexity.

We additionally evaluated whether a random forest model would be more discriminating than logistic regression. Although a marginal improvement in training set AUC was observed, confidence intervals for the test set essentially overlapped. We do not think that the small potential gain in discrimination justifies the increase in model complexity and loss of interpretability. It may be the case that the specific advantages of the forest were not relevant to this dataset. In addition, the strong performance of the logistic regression model yielded little grounds for improvement.

All logistic models appear well calibrated by plot and the unreliability index statistic. This suggests that our models were reasonably resilient against concept drift that might have occurred between the training and test data years (2008–2015 vs. 2016). Notably, the number of asymptomatic patients in our sample is small (*n* = 29), which limits the power of graphical methods and statistical tests to identify calibration errors [43].

While a strength of this study is its access to population-based physician claim and hospital discharge data, the use of single center to construct our cohort is a limitation. The resulting models likely include some practice-style features specific to this study’s jurisdiction. For example, the occasional use of the diagnostic code for demyelinating diseases is potentially specific to our jurisdiction and may not generalize. A second limitation of this study is the use of a test set drawn from the same institution. This limits the generalizability of the study to new populations and should be addressed by external validation using a nationally representative sample in future research. Finally, given restrictions on small cell size, we were not able to analyse classification accuracy for asymptomatic patients with peri-operative stroke. These patients are potentially at highest risk for misclassification by discharge diagnosis and are also the numerator cases for peri-operative complications. We thought it preferable to exclude these patients, rather than assume classification accuracy similar to the general population.

The intended future use case for this algorithm is to support national-scale quality monitoring programs for carotid endarterectomy. In this single-center study we demonstrate proof-of-concept for the use of administrative data for accurate classification of symptomatic status. Future work will need to evaluate the algorithm in nationally representative test and development sets. Ideally, the data set would be enriched with known cases of peri-operative complication, to ensure that the algorithm correctly classifies these high-importance cases.

## Conclusion

Classification of symptomatic status for carotid endarterectomy by discharge diagnosis is insensitive. Logistic regression models combining physician claims and discharge data yield discriminating and well-calibrated models.

## Supplementary Information

**Additional file 1.** List of R packages. Literature review including Supplementary Table 1, Supplementary Tables 2–4, Supplementary Figs. 1 & 2. Annotated statistical code.

## Data Availability

The data that support the findings of this study are available from Population Data BC, but restrictions apply to the availability of these data, which were used under license for the current study, and so are not publicly available. Data are however available from the authors upon reasonable request and with permission of Population Data BC.

## Abbreviations

CEA: Carotid endarterectomy
ICD: International Classification of Diseases
ROC: Receiver operating characteristic curve
AUC: Area under curve (in this context, area under the receiver operating characteristic curve)
LOESS: Locally-estimated scatterplot smoothing

## References

[1] Rothwell PM, Goldstein LB (2004). Carotid endarterectomy for asymptomatic carotid stenosis. Stroke..

[2] Rothwell P, Eliasziw M, Gutnikov S, Warlow C, Barnett H, Collaboration for the CET (2004). Endarterectomy for symptomatic carotid stenosis in relation to clinical subgroups and timing of surgery. Lancet..

[3] Keyhani S, Cheng EM, Hoggatt KJ, Austin PC, Madden E, Hebert PL, Halm EA, Naseri A, Johanning JM, Mowery D (2020). Comparative effectiveness of carotid endarterectomy vs initial medical therapy in patients with asymptomatic carotid stenosis. Jama Neurol.

[4] Howard VJ, Meschia JF, Lal BK, Turan TN, Roubin GS, Brown RD, Voeks JH, Barrett KM, Demaerschalk BM, Huston J (2017). Carotid revascularization and medical management for asymptomatic carotid stenosis: protocol of the CREST-2 clinical trials. Int J Stroke.

[5] Brott TG, Hobson RW, Howard G, Roubin GS, Clark WM, Brooks W, Mackey A, Hill MD, Leimgruber PP, Sheffet AJ (2010). Stenting versus endarterectomy for treatment of carotid artery stenosis. New Engl J Med.

[6] Bensley RP, Yoshida S, Lo RC, Fokkema M, Hamdan AD, Wyers MC, Chaikof EL, Schermerhorn ML (2013). Accuracy of administrative data versus clinical data to evaluate carotid endarterectomy and carotid stenting. J Vasc Surg.

[7] Perler BA, Dardik A, Burleyson GP, Gordon TA, Williams GM (1998). Influence of age and hospital volume on the results of carotid endarterectomy: a statewide analysis of 9918 cases. J Vasc Surg.

[8] Dardik A, Bowman HM, Gordon TA, Hsieh G, Perler BA (2000). Impact of race on the outcome of carotid endarterectomy. Ann Surg.

[9] Matsen SL, Chang DC, Perler BA, Roseborough GS, Williams GM (2006). Trends in the in-hospital stroke rate following carotid endarterectomy in California and Maryland. J Vasc Surg.

[10] McPhee JT, Schanzer A, Messina LM, Eslami MH (2008). Carotid artery stenting has increased rates of postprocedure stroke, death, and resource utilization than does carotid endarterectomy in the United States, 2005. J Vasc Surg.

[11] Giacovelli JK, Egorova N, Dayal R, Gelijns A, McKinsey J, Kent KC (2010). Outcomes of carotid stenting compared with endarterectomy are equivalent in asymptomatic patients and inferior in symptomatic patients. J Vasc Surg.

[12] Eslami MH, McPhee JT, Simons JP, Schanzer A, Messina LM (2011). National trends in utilization and postprocedure outcomes for carotid artery revascularization 2005 to 2007. J Vasc Surg.

[13] Khatri R, Chaudhry SA, Vazquez G, Rodriguez GJ, Hassan AE, Suri MFK, Qureshi AI (2012). Age differential between outcomes of carotid angioplasty and stent placement and carotid endarterectomy in general practice. J Vasc Surg.

[14] Wang FW, Esterbrooks D, Kuo Y-F, Mooss A, Mohiuddin SM, Uretsky BF (2011). Outcomes after carotid artery stenting and endarterectomy in the Medicare population. Stroke..

[15] Qureshi AI, Chaudhry SA, Hussein HM, Majidi S, Khatri R, Rodriguez GJ, Suri MFK (2012). A comparison of outcomes associated with carotid artery stent placement performed within and outside clinical trials in the United States. J Vasc Surg.

[16] Broderick J, Brott T, Kothari R, Miller R, Khoury J, Pancioli A, Gebel J, Mills D, Minneci L, Shukla R (1998). The greater Cincinnati/northern Kentucky stroke study. Stroke..

[17] Fokkema M, Hurks R, Curran T, Bensley RP, Hamdan AD, Wyers MC, Moll FL, Schermerhorn ML (2014). The impact of the present on admission indicator on the accuracy of administrative data for carotid endarterectomy and stenting. J Vasc Surg.

[18] Galiñanes EL, Dombroviskiy VY, Hupp CS, Kruse RL, Vogel TR (2014). Evaluation of readmission rates for carotid endarterectomy versus carotid artery stenting in the US Medicare population. Vasc Endovasc Surg.

[19] Al-Damluji MS, Dharmarajan K, Zhang W, Geary LL, Stilp E, Dardik A, Mena-Hurtado C, Curtis JP (2015). Readmissions after carotid artery revascularization in the Medicare population. J Am Coll Cardiol.

[20] Brinjikji W, El-Sayed AM, Kallmes DF, Lanzino G, Cloft HJ (2015). Racial and insurance based disparities in the treatment of carotid artery stenosis: a study of the Nationwide inpatient sample. J Neurointerv Surg.

[21] Watanabe M, Chaudhry SA, Adil MM, Alqadri SL, Majidi S, Semaan E, Qureshi AI (2015). The effect of atrial fibrillation on outcomes in patients undergoing carotid endarterectomy or stent placement in general practice. J Vasc Surg.

[22] Chaudhry SA, Afzal MR, Kassab A, Hussain SI, Qureshi AI (2016). A new risk index for predicting outcomes among patients undergoing carotid endarterectomy in large administrative datasets. J Stroke Cerebrovasc Dis.

[23] Hussain MA, Mamdani M, Saposnik G, Tu JV, Turkel-Parrella D, Spears J, Al-Omran M (2016). Validation of carotid artery revascularization coding in Ontario health administrative databases. Clin Investigative Med.

[24] Lichtman JH, Jones MR, Leifheit EC, Sheffet AJ, Howard G, Lal BK, Howard VJ, Wang Y, Curtis J, Brott TG (2017). Carotid endarterectomy and carotid artery stenting in the US Medicare population, 1999-2014. Jama..

[25] Reznik M, Kamel H, Gialdini G, Pandya A, Navi BB, Gupta A (2018). Timing of carotid revascularization procedures after ischemic stroke. Stroke..

[26] Salzler GG, Farber A, Rybin DV, Doros G, Siracuse JJ, Eslami MH (2017). The association of carotid revascularization endarterectomy versus stent trial (CREST) and Centers for Medicare and Medicaid Services carotid guideline publication on utilization and outcomes of carotid stenting among “high-risk” patients. J Vasc Surg.

[27] Cole TS, Mezher AW, Catapano JS, Godzik J, Baranoski JF, Nakaji P, Albuquerque FC, Lawton MT, Little AS, Ducruet AF (2020). Nationwide trends in carotid endarterectomy and carotid artery stenting in the post-CREST era. Stroke..

[28] Gupta PK, Pipinos II, Miller WJ, Gupta H, Shetty S, Johanning JM, Longo GM, Lynch TG (2011). A population-based study of risk factors for stroke after carotid endarterectomy using the ACS NSQIP database. J Surg Res.

[29] Shean KE, McCallum JC, Soden PA, Deery SE, Schneider JR, Nolan BW, Rockman CB, Schermerhorn ML (2017). Initiative S for VSVQ. Regional variation in patient selection and treatment for carotid artery disease in the vascular quality initiative. J Vasc Surg.

[30] Columbo JA, Martinez-Camblor P, MacKenzie TA, Kang R, Trooboff SW, Goodney PP, O’Malley AJ (2018). A comparative analysis of long-term mortality after carotid endarterectomy and carotid stenting. J Vasc Surg.

[31] Nejim B, Alshwaily W, Dakour-Aridi H, Locham S, Goodney P, Malas MB (2019). Age modifies the efficacy and safety of carotid artery revascularization procedures. J Vasc Surg.

[32] Aridi HD, Paracha N, Nejim B, Locham S, Malas MB (2017). Anesthetic type and hospital outcomes after carotid endarterectomy from the vascular quality initiative database. J Vasc Surg.

[33] British Columbia Ministry of Health [creator]. Medical Services Plan (MSP) Payment Information File [Internet]. 2019;Available from: http://www.popdata.bc.ca/data

[34] British Columbia Ministry of Health [creator]. Consolidation file (MSP Registration & Premium Billing) [Internet]. 2019;Available from: http://www.popdata.bc.ca/data

[35] Canadian Institute for Health Information [creator]. Discharge abstract database (hospital separations) [Internet]. 2019;Available from: http://www.popdata.bc.ca/data

[36] Hu W. Diagnostic codes in MSP claim data: summary report. Medi Serv Plan. 1996.

[37] Kuhn M, Johnson K (2013). Applied predictive modeling.

[38] Brown LD, Cai TT, DasGupta A (2001). Interval estimation for a binomial proportion. Stat Sci.

[39] Kuhn M, Wickham H. Tidymodels: a collection of packages for modeling and machine learning using tidyverse principles. [Internet]. 2020;Available from: https://www.tidymodels.org

[40] Friedman J, Hastie T, Tibshirani R (2010). Regularization paths for generalized linear models via coordinate descent. J Stat Softw.

[41] Wright MN, Ziegler A. Ranger: a fast implementation of random forests for high dimensional data in C++ and R. J Stat Softw. 2017;77(1):1–17.

[42] Austin PC, Steyerberg EW (2014). Graphical assessment of internal and external calibration of logistic regression models by using loess smoothers. Stat Med.

[43] Van Calster B, McLernon DJ, van Smeden M, Wynants L, Steyerberg EW, Bossuyt P (2019). Calibration: the Achilles heel of predictive analytics. BMC Med.

