# Supplementary material for: Accurate classification of carotid endarterectomy indication using physician claims and hospital discharge data
Source: BMC Health Serv Res. 2022 Mar 22;22:379. doi: 10.1186/s12913-022-07614-1 (PMC8941812; doi:10.1186/s12913-022-07614-1)
Supplement: Supplementary file 1 — Additional file 1. List of R packages. Literature review including Supplementary Table 1, Supplementary Tables 2–4, Supplementary Figs. 1 & 2. Annotated statistical code. [file 12913_2022_7614_MOESM1_ESM.pdf]

Accurate classification of carotid endarterectomy indication using physician claims and hospital discharge data – Data Supplement

LIST OF R PACKAGES

R version 3.6.1 (2019-07-05)

Platform: x86_64-w64-mingw32/x64 (64-bit), Windows 10 x64 (build 18362)

Base: stats, graphics, grDevices, utils, datasets, methods, base

Attached: furrr_0.2.1, future_1.19.1, pROC_1.17.0.1, icd.data_1.0, rules_0.0.2, yardstick_0.0.7, workflows_0.2.0, tune_0.1.1, rsample_0.0.7, recipes_0.1.13, parsnip_0.1.3, modeldata_0.0.2, infer_0.5.3, dials_0.0.9, scales_1.1.1, broom_0.7.0, tidymodels_0.1.1, forcats_0.5.1, stringr_1.4.0, dplyr_1.0.4, purrr_0.3.4, readr_1.4.0, tidyr_1.1.2, tibble_3.0.6, ggplot2_3.3.3, tidyverse_1.3.0, magrittr_2.0.1, here_0.1

Loaded via namespace: via namespace: colorspace_2.0-0, ellipsis_0.3.1, class_7.3-15, rprojroot_2.0.2, fs_1.5.0, rstudioapi_0.13, listenv_0.8.0, farver_2.0.3, prodlim_2018.04.18, lubridate_1.7.9.2, ranger_0.12.1, xml2_1.3.2, codetools_0.2-16, splines_3.6.1, knitr_1.30, jsonlite_1.7.2, caret_6.0-86, BlandAltmanLeh_0.3.1, dbplyr_1.4.2, compiler_3.6.1, httr_1.4.2, backports_1.2.0, assertthat_0.2.1, Matrix_1.2-17, cli_2.3.0, htmltools_0.5.1.1, tools_3.6.1, gtable_0.3.0, glue_1.4.2, reshape2_1.4.3, Rcpp_1.0.6, cellranger_1.1.0, DiceDesign_1.8-1, vctrs_0.3.6, nlme_3.1-140, iterators_1.0.10, timeDate_3043.102, gower_0.2.1, xfun_0.20, globals_0.13.1, rvest_0.3.5, lifecycle_0.2.0, MASS_7.3-53, ipred_0.9-9, hms_1.0.0, parallel_3.6.1, yaml_2.2.1, rpart_4.1-15, stringi_1.5.3, highr_0.8, foreach_1.4.4, lhs_1.0.1, hardhat_0.1.4, shape_1.4.4, lava_1.6.5, epiR_1.0-2, rlang_0.4.10, pkgconfig_2.0.3, evaluate_0.14, lattice_0.20-38, labeling_0.4.2, tidyselect_1.1.0, plyr_1.8.4, R6_2.5.0, generics_0.1.0, DBI_1.1.0, pillar_1.4.7, haven_2.2.0, withr_2.4.1, mgcv_1.8-28, survival_3.2-7, nnet_7.3-12, modelr_0.1.5, crayon_1.4.1, rmarkdown_2.5, grid_3.6.1, readxl_1.3.1, data.table_1.13.6, ModelMetrics_1.2.2.2, reprex_0.3.0, digest_0.6.27, stats4_3.6.1, glmnet_4.1, GPfit_1.0-8, munsell_0.5.0, viridisLite_0.3.0, BiasedUrn_1.07

LITERATURE REVIEW

Search strategy

Last executed: 2020-09-19

1. Endarterectomy, Carotid/

2. Time-to-Treatment/

3. limit 2 to (abstracts and English language and yr="2013 -Current")

4. 1 and 3

5. Time Factors/

6. limit 5 to (abstracts and English language and yr="2000 - 2013")

7. 1 and 6

8. Time Factors/

9. limit 8 to (abstracts and English language and yr="2013 -Current")

10. 1 and 9

11. 4 or 7 or 10

12. Quality Improvement/

13. Endarterectomy, Carotid/sn [Statistics & Numerical Data]

14. 1 and 12

15. 11 or 13 or 14

16. limit 15 to yr="2000 -Current"

17. limit 16 to (abstracts and English language)

18. from 11 keep 6,8

19. from 17 keep 7,37-38,52,60,65,107,109-110,118

20. from 17 keep 159,173,188-189,215,235,299,312,345,348,353,365,376,381

21. from 17 keep 421,440,450

22. from 17 keep 478,490,506,516,520,531-532,540,567-568

23. from 17 keep 630,648,651,725,741,839,872

24. 18 or 19 or 20 or 21 or 22 or 23

25. delay*.mp.

26. 1 and 25

27. 26 not 17

28. from 27 keep 34,42,50,67

29. administr*.mp.

30. (1 and 29) not 17

31. limit 30 to (abstracts and english language and yr="2004 -Current")

32. symptom*.mp. [mp=title, abstract, original title, name of substance word, subject heading word, keyword heading word, protocol supplementary concept word, rare disease supplementary concept word, unique identifier, synonyms]

33. day*.mp. [mp=title, abstract, original title, name of substance word, subject heading word, keyword heading word, protocol supplementary concept word, rare disease supplementary concept word, unique identifier, synonyms]

34. week*.mp. [mp=title, abstract, original title, name of substance word, subject heading word, keyword heading word, protocol supplementary concept word, rare disease supplementary concept word, unique identifier, synonyms]

35. (1 and 32 and (33 or 34)) not (17 or 31 or 27)

36. limit 35 to (abstracts and english language and yr="2004 -Current")

37. from 31 keep 9,11,16,24,29

38. from 36 keep 5,60,75,81,85,109

39. from 37 keep 1-2,4-5

40. from 36 keep 152

41. 28 or 37 or 38 or 39 or 40 or 24

Literature summary

Supplementary Table 1. Summary of administrative data (A) and registry (R) studies of carotid artery revascularization including surgical indication.

#	A/R	Data source	Symptomatic status		
			Approach	Validation	Prevalence
7	A	Hospital database (Johns Hopkins) for method, state database (Maryland) for analysis	Not stated		
8	A	State database (Maryland)	ICD-9-CM 342, 438, 435, 781.4, 362.34, 368.12 as 'reason for surgery' (unclear where coded)	Attributes review of Charlson comorbidity index[45]	17.9%
9	A	State databases (Maryland, California)	Same approach as [8]	States validated against Johns Hopkins cohort, but no statistics reported; see letter to editor and response	15.2%
10	A	Nationwide Inpatient Sample (2005)	Procedural diagnosis 433.11 or secondary diagnosis of stroke / TIA (codes not specified)	Not stated	7.9%
11	A	State databases (New York, California)	ICD-9-CM 362.3, 362.84, 433.11, 433.31, 434.11, 434.91, 435.8, 435.9, 434.01	Development using hospital database, but Not stated set and no statistics reported	11.0%

#	A/R	Data source	Symptomatic status		
			Approach	Validation	Prevalence
12	A	Nationwide Inpatient Sample	ICD-9-CM 433.11 or secondary diagnosis of stroke, TIA (codes not specified)	Not stated, cites coding manual	8.2%
13	A	Nationwide Inpatient Sample (2005-2008)	Same approach as[10]	Not stated	5%
14	A	CMS Provider Analysis Review & Denominator (2003-2006)	Primary diagnosis 433.11 or secondary diagnosis (ICD-9 codes 342:34200 to 34202, 3421, 34210, 34211, 34212, 34280 to 34282, 34290 to 34292, or 438: 4380, 43810 to 43812, 43819 to 43822, 43830 to 43832, 43840 to 43842, 43850 to 43853, 4386, 4387 43881 to 43885, 43889, 4389), 435 or 781.4, or 362.34 or 368.12	Not stated. Cites[9] but method is considerably different	12.5%
15	A	Nationwide Inpatient Sample (2005-2009)	Same approach as [10]	Not stated. Cites[16], but this is a study of stroke/TIA not revascularization	Not reported (used as covariate in analysis)

#	A/R	Data source	Symptomatic status		
			Approach	Validation	Prevalence
17	A	State databases (California 2005-2008, New York 2008, New Jersey 2008)	Three methods: (A) Unspecified diagnosis codes for stroke, TIA, amaurosis fugax, (B) A without stroke, (POA) A with stroke with POA indicator	Not stated. Agreement between methods reported, but no gold standard.	(A) 15.9%, (B) 7.1%, (POA) 15.4%
18	A	CMS Medicare Provider Analysis and Review (2005-2009)	ICD-9-CM 362.34, 435.X, 781.4 in any diagnosis position	Not stated	2.7%
19	A	Medicare fee-for-service claims (2009-2011)	362.3[0–7], 362.84, 433.11, 433.31, 434.01, 434.91, 435.[0–3,8,9], and 781.4 [2,4,5]	Not stated (reports 'previously reported in literature' but no citations)	10%
20	A	Nationwide Inpatient Sample (2005-2011)	Asymptomatic if diagnosis code 433.10, 433.30; symptomatic if 433.11, 433.31 (unclear how -/- cases handled)	Not stated	7.1-9% by year of study
21	A	Nationwide Inpatient Sample (2005-2009)	Same approach as [10]	Not stated	6%
22	A	Nationwide Inpatient Sample (2005-2006)	Same approach as [10]	Not stated	Not reported (covariate for modeling)

#	A/R	Data source	Symptomatic status		
			Approach	Validation	Prevalence
[24]	A	CMS Medicare Denominator (1999-2014)	433.11, 433.31,<br>434.01, 434.11, or 434.91; or secondary diagnosis 342.xx, 438.xx, 435.x, 781.4, 362.34 or 368.12	Reports reference for [14] but method includes 434.X1 codes that were not in that study	12.5%
[25]	A	Healthcare Cost and Utilization database (2005-2013)	Hospitalization for ischemic stroke < 90 d 433.X1, 434.X1, 436 without V57, 430.X, 431.X, 800-804, 850-854	Reports reference, but not applicable	N/A
[26]	A	Nationwide Inpatient Sample (2005-2011)	Same method as [10]	Not stated	4.8%
[27]	A	Nationwide Readmissions Database (2010-2015)	Extensive set of 5-digit ICD-9 codes encompassing ischemic diagnoses and symptoms	Not stated	21.0% CEA<br>30.1% CAS
[28]	R	National Surgical Quality Improvement Program (2007-2008)	Reported history of stroke or TIA	Not stated	43.5%
[6]	A/R	National Surgical Quality Improvement Program and hospital administrative discharge data (2005-2011)	ICD-9-CM 435.X, 781.4, V12.54, 362.3X, 368.12, 433.11, 433.31, 433.91, 434.01, 434.11, 434.91; NSQIP history of stroke / TIA	Physician chart review vs. discharge abstracts (n=1342) sensitivity 36.6, specificity 93.1, PPV 73.2, NPV 74; vs. NSQIP (n=392) sensitivity 91.6%, PPV 63.0%	17% administrative data, 34% chart review, 44% NSQIP

#	A/R	Data source	Symptomatic status		
			Approach	Validation	Prevalence
[29]	R	Vascular Quality Initiative (2009-2015)	Declared history of ipsilateral stroke, TIA, or retinal ischemia	Not stated	44% CEA<br>64% CAS
[30]	R	Vascular Quality Initiative (2003-2013)	Not stated	Not stated	38.3% CEA<br>48.3% CAS
[31]	R	Vascular Quality Initiative (2005-2017)	Declared history of ipsilateral stroke, TIA, or retinal ischemia	Not stated	34.1% CEA<br>44.5% CAS
[32]	R	Vascular Quality Initiative (2003-2017)	Not stated	Not stated	30.7% CEA

SUPPLEMENTARY TABLES

Supplementary Table 2. Candidate cluster definitions for physician claims diagnoses, with provider-type dyads

Diagnosis cluster	ICD 9 (3 digit)	Provider type
**Retinal (ophthalmology / optometry)**	362 or 368	Ophthalmology or optometry
**Retinal (neurology)**	362 or 368	Neurology
**Retinal (other)**	362 or 368	All others
**Stroke (neurology)**	434 or 436	Neurology
**Stroke (other)**	434 or 436	All others
**TIA (neurology)**	435	Neurology
**TIA (other)**	435	All others
**Stenosis (neurology)**	433	Neurology
**Stenosis (other)**	433	All others
**Symptom**	781 or 341	All

Supplementary Table 3. Candidate cluster definitions for service items, by cluster set (full, partial, and enhanced).

Service cluster	Cluster set	Fee codes
**After hours visit**	Full and enhanced	1200, 1201, 1202, 1205, 1206, 1207, 1210, 1211, 1212, 1215, 1216, 1217
**Anesthesia visit**	Full and enhanced	1080, 1164, 1165, 1169, 1172, 1173, 1174, 1175, 1176, 1177, 1178, 1179, 1180, 1181, 1192
**Holter**	All	33047, 33048, 33049, 33062, 33063, 33065, 33069, 33092
**Echocardiogram**	All	8679, 33091
**Cardiology visit**	Full and enhanced	33006, 33007, 33008, 33010, 33012, 33110, 33112, 33114
**Carotid CTA**	All	100001 (generated value based on coincidental CT head and CT body)
**Carotid ultrasound**	All	8676
**Emergency visit**	All	1810, 1811, 1812, 1813, 1821, 1822, 1823, 1831, 1832, 1833, 1841, 1842, 1843, 96801, 96802, 96803, 96804, 96805, 96811, 96812, 96813, 96814, 96815, 96821, 96822, 96823, 96824, 96825
**Internal medicine visit**	Full and enhanced	310, 311, 312, 314, 32370, 32372, 32271
**Head CT**	All	8690, 8691, 8692
**Neurology visit**	All	406, 407, 408, 410, 411, 40410, 40411
**Optometry / ophthalmology visit**	All	2014 : 2049, 2899
**Optometry / ophthalmology exam**	All	2005, 2007, 2008, 2009, 2010, 2011, 2012, 22007, 22008, 22010, 22011
**Out of office visit**	Enhanced	12200, 12201, 12210, 12220, 13200, 13201, 13210, 13220, 15200,15201, 15210, 15220, 16200, 16201, 16210, 16220, 17220, 17201, 17210, 17220, 18200, 18201, 18210, 18220
**Stroke visit**	All	441, 442, 443, 444, 40441, 40442, 40442, 40444

Service cluster	Cluster set	Fee codes
**Cardiac stress test**	Enhanced	33034, 33035, 33036, 95062, 95063

Supplementary Table 4. Test set calibration statistics based on logistic recalibration $Logit(Y)=a+b_L*L$, where L denotes the candidate linear predictor model. The unreliability index evaluates simultaneously H_0: a = 0, b_L = 1 using a 2-df chi-square test.

	**$Logistic_{HOSP}$**	**$Logistic_{DX}$**	**$Logistic_{ALL}$**	**$Forest_{ALL}$**
**Intercept**	-0.32	-0.22	-0.25	-0.57
**Slope**	1.36	0.84	0.95	1.74
**Unreliability p-value**	0.523	0.348	0.680	0.054

Supplementary figures

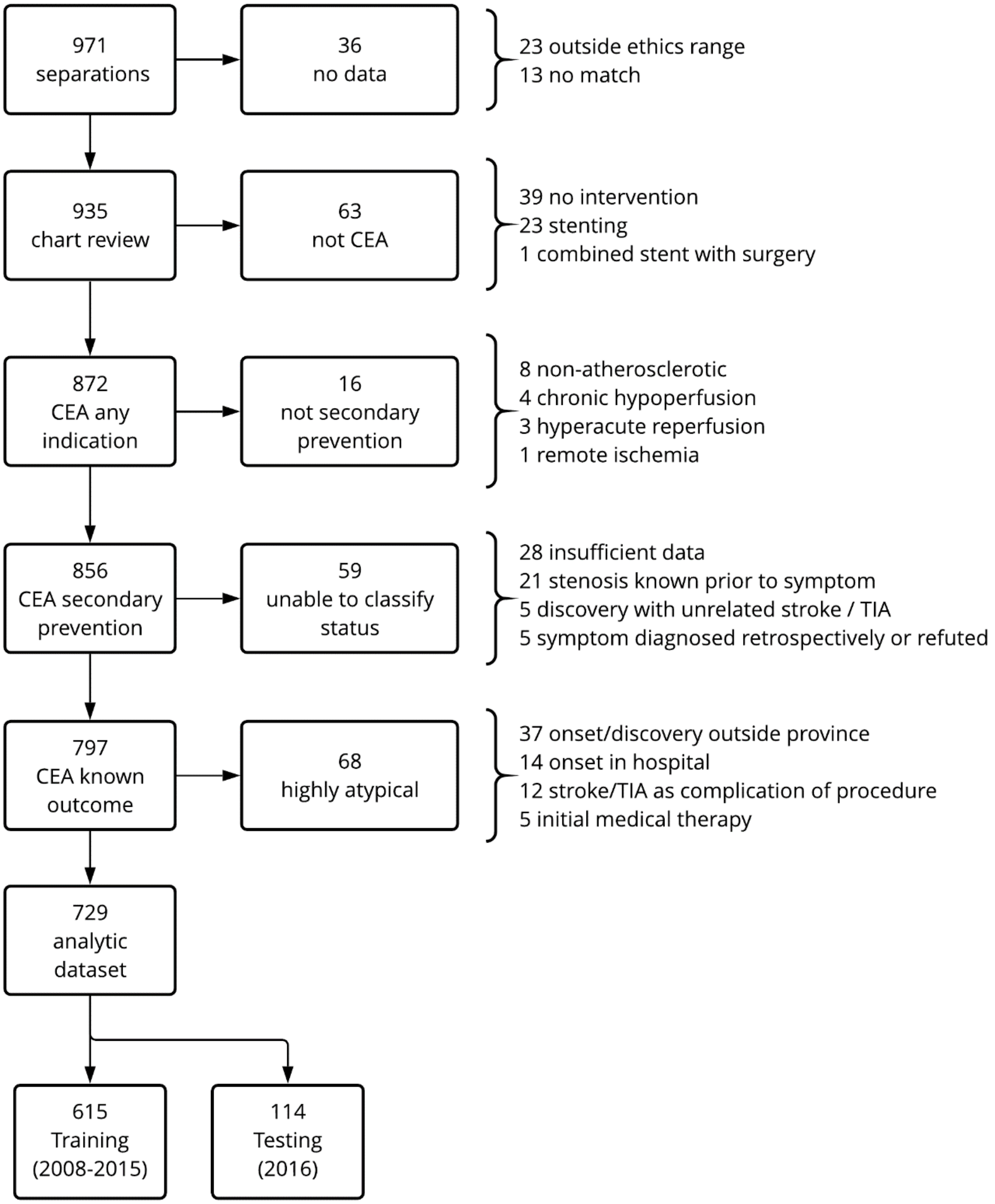

Supplementary Figure 1. Participant flow diagram, Vancouver Canada, 2008-2016.

Feature extraction for physician claims data

Physician claims data set

ID	Date (Days before surgery)	Fee item	Description	Diagnosis	Provider	Specialty
1	2020-02-24 (8)	441	Neurology consult	435	104	Neurology
1	2020-02-28 (4)	442	Neurology follow up	434	104	Neurology
1	2020-02-28 (4)	442	Neurology follow up	434	105	Neurology

Step 1 - Clustering. The claims database offers two types of data that might be relevant to classification - diagnoses and services. We can apply conceptually meaningfully clusters to reduce the number of categories. With reference to our sample data, we have defined a set of diagnosis * specialty and a set of fee item clusters for neurology.

Cluster (dx)	Diagnosis	Specialty
d_tia_neuro	435	Neurology
d_stroke_neuro	434	Neurology
d_stenosis_neuro	433	Neurology

Cluster (fee)	Fee item
f_neurology_visit	441
f_neurology_visit	442
f_neurology_visit	443

Step 2 - Reduce. After clustering, reduction can be applied to remove conceptual duplicates, such as visits to the same provider, or visits on the same day. With reference to our sample data, if we were to reduce diagnosis by provider number, we would keep only the most recent row for provider 104. If we were to reduce fee items by service date, we would discard one of the two neurology visits from 2020-02-28 (arbitrarily, we keep the last row from the set).

ID	Days before	Cluster (dx)	Provider
1	8	d_tia_neuro	104
1	4	d_stroke_neuro	104
1	4	d_stroke_neuro	105

ID	Days before	Cluster (fee)	Provider
1	8	f_neurology_visit	104
1	4	f_neurology_visit	104
1	4	f_neurology_visit	105

Step 3 - Weight. Services that are closer in time to the surgery date might be more predictive than remote services. We can define weight functions, like a linear decay, to capture this. With refence to our sample data, we have applied a 1% per day linear decay to diagnosis and a simple threshold for fee items. All weights are defined to return values between 0 and 1.

ID	Weight	Cluster (dx)	Provider
1	0.96	d_stroke_neuro	104
1	0.96	d_stroke_neuro	105

ID	Weight	Cluster (fee)	Provider
1	1	f_neurology_visit	104
1	1	f_neurology_visit	105

Step 4 - Summarize. Finally, a summarizing function needs to be applied to reduce feature tables to a single row per participant. For our sample data we have used a max value for diagnosis and a sum for fee items. This is combined with a pivot operation to create one row per participant.

ID	d_stroke_neuro	f_neurology_visit
1	0.96 (max)	2 (sum)

Supplementary Figure 2. Feature extraction for physician claims data, illustrating clustering, reduction, weighting, and summarizing functions applied to sample data.

SUPPLEMENTAL REFERENCES

References 1-44 are included in the main manuscript.

45. Romano P, Roos LL, Jollis JG. Adapting a clinical comorbidity index for use with ICD-9-CM administrative data: differing perspectives. *J Clin Epidemiol*. 1993;46:1075–9.
